# The Practical Utility of Imidazolium Hydrogen Sulfate Ionic Liquid in Fabrication of Lignin-Based Spheres: Structure Characteristic and Antibacterial Activity

**DOI:** 10.3389/fchem.2022.946665

**Published:** 2022-07-06

**Authors:** Małgorzata Stanisz, Łukasz Klapiszewski, Anna Dobrowolska, Adam Piasecki, Katarzyna Czaczyk, Teofil Jesionowski

**Affiliations:** ^1^ Institute of Chemical Technology and Engineering, Faculty of Chemical Technology, Poznan University of Technology, Poznan, Poland; ^2^ Department of Biotechnology and Food Microbiology, Poznan University of Life Sciences, Poznan, Poland; ^3^ Institute of Materials Science and Engineering, Faculty of Materials Engineering and Technical Physics, Poznan University of Technology, Poznan, Poland

**Keywords:** spherical particles, lignin, ionic liquids, structural properties, antibacterial properties

## Abstract

In this study, lignin-based spherical particles (Lig-IL) with the use of 1-(propoxymethyl)-1H-imidazolium hydrogen sulfate were prepared in different biopolymer and ionic liquid (IL) weight ratios. The application of IL during the preparation of spherical particles is an innovative method, which may be beneficial for further applications. The particles were obtained with the use of the soft-templating method and their chemical, structural and morphological characterization was performed. The spherical shape of products and their size (91–615 nm) was confirmed with the use of scanning electron microscopy (SEM) images and the particle size distribution results. The attenuated total reflectance-Fourier transform infrared (ATR-FTIR) spectra were analyzed to identify functional groups of all precursors and produced material and it was confirmed, that all materials exhibit characteristic hydroxyl and carboxylic groups, but the presence of carbonyl group was detected. Moreover, the zeta potential analysis was performed to evaluate the electrokinetic behavior of obtained materials. It was confirmed, that all materials are colloidally stable in pH above 4. Produced lignin-based spherical particles were used for evaluation of their antibacterial properties. Particles were tested against *Staphylococcus aureus* (*S. aureus*), a gram-positive bacterium, and *Escherichia coli* (*E. coli*), a gram-negative one. It was observed, that only the material with the highest addition of IL showed the antibacterial properties against both strains. A reduction of 50% in the number of microorganisms was observed for particles with the addition of hydrogen sulfate ionic liquid in a 1:1 ratio after 1 h. However, all prepared materials exhibited the antibacterial activity against a gram-positive bacterium.

## 1 Introduction

Lignin is a biopolymer, which is a by-product of the cellulose and paper industry. It has been mostly used as a heating source, because the structure of lignin is complex with a high molecular weight (Li C. et al., 2015; Li H. et al., 2015; Pang Y. et al., 2020). It is composed mostly of three basic units including hydroxyphenyl, guaiacyl, and syringyl structures (Graichen et al., 2017). Moreover, the microstructure is distributed randomly, which means, that the specific parts may vary depending on different factors including the extraction method of biopolymer (Afewerki et al., 2020; Gan et al., 2020). One of the greatest advantages of lignin is the presence of a variety of functional groups, which makes the biopolymer an amphiphilic material (Schutyser et al., 2018). There are a variety of functional groups in its structure including hydroxyl, carboxylic, and carbonyl ones, which are essential for conducting modification of its structure as well as polymerization (Becker and Wittmann, 2019). Lignin possesses a variety of interesting properties including low cost, biodegradability (Sun et al., 2016), and biocompatibility (Bizmark et al., 2020); therefore, it may be used for 3D printing (Tanase-Opedal et al., 2019; Sugiarto et al., 2022), epoxy resins (Nisha et al., 2019; Zhang et al., 2020), purification of water treatment and biomedical applications (Pishnamazi et al., 2018; Pishnamazi et al., 2019a; Pishnamazi et al., 2019b). Lignin also exhibits antioxidant properties due to the presence of methoxy and phenolic hydroxyl functional groups and it can also serve as an antioxidant (Aadil et al., 2018; You et al., 2020; Kumar et al., 2021). It is due to the presence of methyl group in the gamma position and C-C double bond, which are localized in the side chain, in the alpha and beta positions. The membrane of the cell may be destroyed and the content of a cell may be released (Aadil et al., 2018). Due to its antibacterial and antioxidant properties, it may be used in medicine, cosmetics, and the food industry (Abdullah et al., 2020; Lizundia et al., 2020).

Recently, it has been observed that lignin-based micro and nanostructures showed an increased interest among researchers (Caruso et al., 1998; Stanisz et al., 2020). Lignin spheres exhibit increased surface, regular shape with different morphology including core-shell and hollow particles (Jiang et al., 2019; Sipponen et al., 2019). Spheres are more stable and uniform; therefore, they may be used as a stabilizer for emulsions (Dickinson, 2017; Bizmark et al., 2020). Preparation of spherical particles with the use of lignin may be an alternative to commonly used materials, especially to replace toxic nanoparticles and for enhancing ultraviolet (UV) properties, and as sorbents for pollutants (Wang et al., 2021; Zhou et al., 2021). Moreover, it may be used as carriers for drug and fertilizer delivery as well as for food packaging (Pang T. et al., 2020; Li et al., 2021). During the preparation of lignin-based spherical particles, some important properties should be fulfilled to prepare materials for industrial applications. Lu *et al.* presented nanospheres with smooth surfaces and structures and regular shapes. Moreover, by adjusting the preparation procedure, they were able to obtain particles with specific shapes and morphology including hollow spheres (Lu et al., 2021). The addition of surfactants may also be beneficial while preparing spheres. Lignin-based spherical particles were prepared hydrothermally and were applied for reduction and support during the synthesis of palladium nanoparticles. The surfactants improved the morphology and homogeneity of the obtained spherical particles. The application of lignin spheres for the synthesis of palladium particles is more eco-friendly than standard methods and results in improvement of catalytic activity (Pang et al., 2021). Nisha et al. (2019) used the triethylammonium hydrogen sulfate ionic liquid to modify the surface of lignin particles in micro and nanoscale for the preparation of biopolymer-based filler in an epoxy matrix. The addition of modified lignin increased the toughness, tensile, and flexural strength of the epoxy composite. Liu et al. (2019) dissolved commercial alkali lignin in a variety of imidazole ionic liquids including 1-amyl-3-methylimidazolium acetate, 1-butyl-3-methylimidazolium acetate, and 1-ethyl-3-methylimidazolium acetate. The obtained particles were small (52–210 nm) and although the aromatic structure of the biopolymer was preserved, the molecular weight of lignin nanoparticles was lower compared to untreated alkali lignin. Lignin-based spherical particles have started to be used for their antimicrobial application. Gabov et al. (2017) prepared cellulose-lignin spheres and tested them with the use of *Escheria coli* and *Staphylococcus aureus* bacteria. It was indicated, that the growth of bacteria was only inhibited for *S. aureus* and the antibacterial properties against gram-positive bacteria increased with the increased amount of lignin. On the other hand, Mili et al. (2021) presented nanolignin particles which were prepared from the bamboo-derived macro-rich fraction with the use of an ultrasonication procedure. They were tested again with gram-positive and gram-negative bacteria and the antibacterial activity of prepared material and UV blocking effect was observed; therefore, the nanolignin material may be used as an antioxidant and antibacterial agent. Li et al. (2020) prepared phase change microcapsules which consisted of polyurea, n-eicosane, and the system was decorated with lignin spherical particles, which acted as a Pickering stabilizer and a reducing agent for silver. Finally, the particles were prepared with a core-shell morphology and great antibacterial activity. Sánchez-Hernández et al. (2022) produced lignin-chitosan-based nanoparticles which were loaded with natural bioactive products and they were tested against *Diplodia seriata*, *Xylophilus ampelinus*, and *Pseudomonas syringae pv. syringae* and the antibacterial activity was observed with good efficiency. Ali et al. (2022) prepared lignin nanospheres from cotton by-products by the organosolv technique. It was observed that obtained materials exhibited great antibacterial properties, several times higher than conventional antibiotics. The obtained materials may be in the future incorporated into the medical equipment including bandages or bed sheets. Lignin-based spherical particles can be also incorporated into hydrogels for antibacterial purposes. Deng et al. (2021) synthesized lignin spheres and decorated them with silver nanoparticles. Lignin was mostly used for the reduction of silver material as well as for supporting the construction of hydrogel.

In this work, our main focus was to prepare lignin-based spherical particles with the use of 1-(propoxymethyl)-1H-imidazolium hydrogen sulfate in a variety of biopolymer and IL ratios. In this method, ionic liquid served as a soft template for the fabrication of spheres. Hydrogen sulfate ionic liquid was used for the first time for the preparation of spherical particles. This is also a novel method for the synthesis of samples because synthetic surfactants are used mostly for this procedure. During the research, an attempt to minimize the use of synthetic substances was performed to reduce the possible environmental pollution. Moreover, ionic liquids are known for their recycling ability, therefore they can be collected after the synthesis and reused for another procedure (Passos et al., 2014; Xin and Hao, 2014). The detailed characteristic was performed including the particles’ size, morphology, and porous structure. Moreover, characteristic bands were evaluated with the use of ATR-FTIR spectroscopy. The electrokinetic stability and elemental compositions were also performed to confirm the correctness of the proposed methods including the combination of lignin with ionic liquid. The obtained materials were also tested for their antibacterial properties against *Staphylococcus aureus*, a gram-positive bacterium, and against *Escherichia coli*, a gram-negative one.

## 2 Experimental

### 2.1 Materials

Kraft lignin, imidazole, chloromethyl propyl ether, formaldehyde, alcohols (ethanol, 1-butanol, cyclohexanol, and benzyl alcohol), and the solvents acetonitrile and heptane were received from Sigma Aldrich, Steinheim, Germany.

### 2.2 Preparation of Ionic Liquid

**FIGURE 1:**
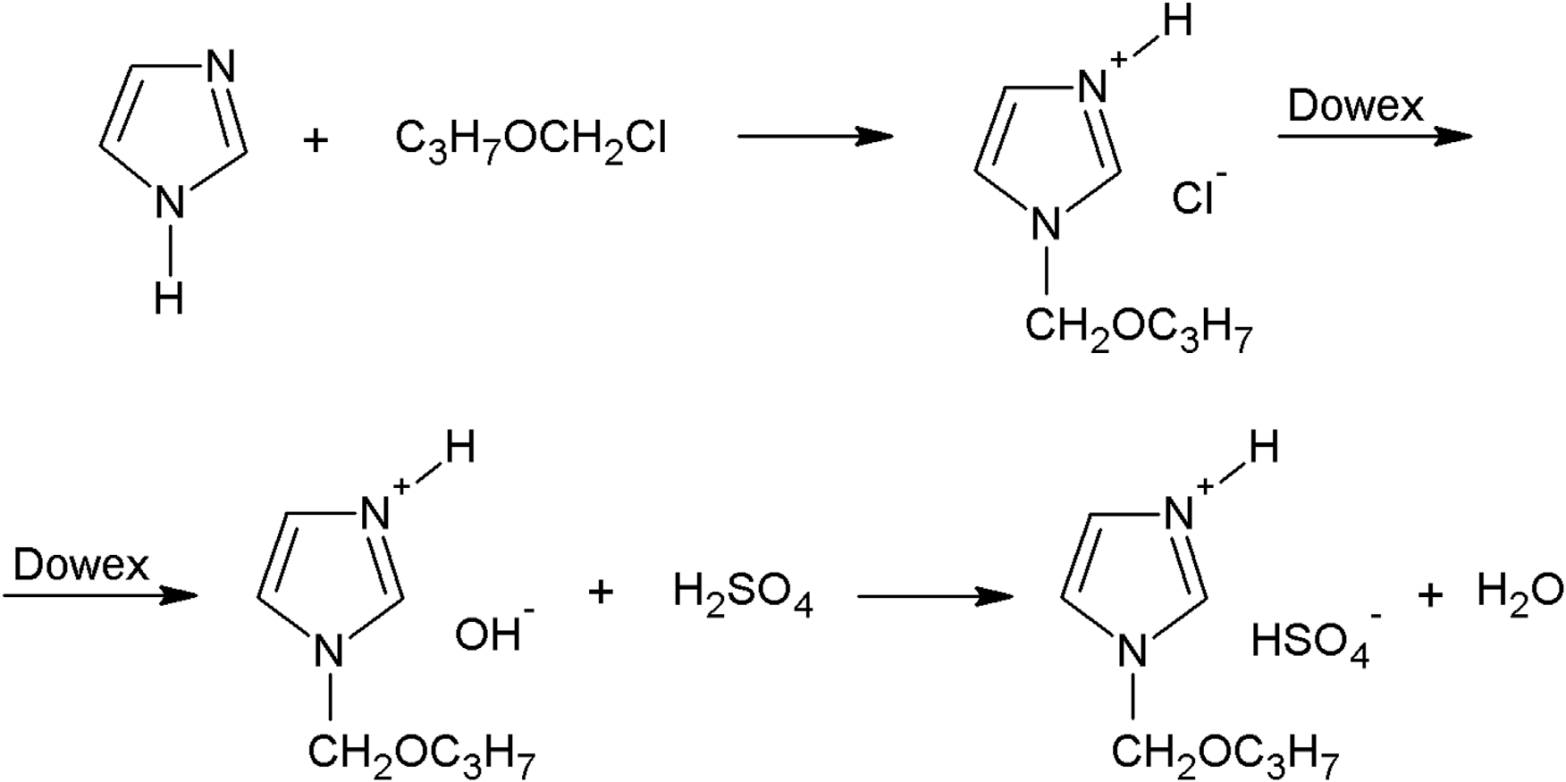
Synthesis of 1-(propoxymethyl)-1H-imidazolium hydrogen sulfate ionic liquid.

The synthesis of acidic imidazolium ionic liquid consisted of three steps. In the first step, imidazolium chloride was obtained by a reaction of imidazole with chloromethyl propyl ether (see Figure 1). Chloromethyl propyl ether was obtained by passing HCl gas through a mixture of formaldehyde and the appropriate alcohol, according to the procedure previously described (Klapiszewski et al., 2018a). The final chloromethyl propyl ether was distilled twice under reduced pressure. The yield of this reaction was about 80%. The 1-(propoxymethyl) imidazolium chloride was prepared as follows. An anhydrous solution of 0.1 M of imidazole in acetonitrile was added to 0.11 M of the chloromethyl propyl ether in acetonitrile. The reaction was carried out for 1 h at 50°C and then the solvent was evaporated in a vacuum. Chloromethyl propyl ether readily hydrolyzed in the presence of small amounts of water to form HCl, which in turn enabled the formation of 1-(propoxymethyl) imidazolium hydrochloride. Separation of the quaternization product and 1-(propoxymethyl) imidazolium hydrochloride is practically impossible. For this reason, quaternization reactions with chloromethyl propyl ether were conducted under strictly anhydrous conditions. The quaternization reaction product was purified by extraction with heptane at 70°C. The yield of the hygroscopic product 1-(propoxymethyl) imidazolium chloride was about 90%. In the second step, chloride anions in imidazolium chloride were exchanged for hydroxide anions, with the use of an ion-exchange resin (Dowex Monosphere 550 A UPW OH form resin) (see Figure 1). The imidazolium hydroxide was prepared as follows. 0.07 M of imidazolium chloride was dissolved in water and passed through a column filled with ion exchange resin. In the third step, the obtained imidazolium hydroxide was immediately subjected to a reaction with sulfuric acid and the corresponding imidazolium hydrogen sulfate was obtained (see Figure 1). Water was evaporated in a vacuum. 1-(propoxymethyl)-1H-imidazolium hydrogen sulfate was synthesized. The yield was 99.5%. We believe, that the presence of additional hydrogen as well as an aliphatic chain may enhance the modification of lignin and therefore form the spherical particles, therefore we have chosen to synthesize the ionic liquid rather than use the commercial one.

### 2.3 Preparation of Lig-IL Materials

For this procedure, kraft lignin was combined with 1-(propoxymethyl)-1H-imidazolium hydrogen sulfate for the preparation of biopolymer-based spherical particles with the use of the soft templating method. The lignin-based particles were obtained with the use of precursors in different weight ratios namely 1:1, 4:3, 2:1, 4:1, and 10:1. Specific amounts of kraft lignin and hydrogen sulfate ionic liquid were dispersed in 50 ml of ethyl alcohol in separate flasks. Then it was mixed for 2 h. To obtain a clear solution, the mixture was filtered in order to obtain only the fraction of lignin which is soluble in ethyl alcohol. The 500 ml of deionized water was added into the mixture of kraft lignin and ionic liquid with the use of a peristaltic pump to precipitate lignin-based spherical particles. Finally, the products were separated from the mixture using a Sartorius Stedim system.

### 2.4 Characterization of Lignin-Based Spherical Particles

Scanning electron microscope (SEM) images were performed for characterization of surface morphology and microstructure of lignin-based spherical particles including the shape and size of individual particles. Mira-3 scanning electron microscope (Tescan, Brno, Czech Republic) was used for the preparation of SEM images. Moreover, the determination of dispersion properties including particle size distribution and polydispersity indices was performed with the use of a Zetasizer Nano ZS instrument (Malvern Instruments Ltd., Malvern, UK). The measurements may be taken for particles in sizes ranging from 0.6 to 6,000 nm and it employs a non-invasive backscattering method (NIBS). For sample preparation, the specified amount of prepared material is sonicated in a Sonic-3 ultrasonic bath (Polsonic, Warszawa, Poland) for 10 min to form a solid dispersion in a chosen media.

Description of porous structure properties was determined with the use of parameters including BET surface area (*A*_*BET*_), the total volume of pores (*V*_*p*_), and mean size of pores (*S*_*p*_). ASAP 2020 apparatus (Micrometeritics Instrument Co. Norcross, United States) was used for measurements. The total volume and mean size of pores were determined with the use Barret–Joyner–Halenda (BJH) algorithm and with the use of the Brunauer–Emmett–Teller method, the surface area was determined. The amount of nitrogen adsorbed on the pore surface under the relative pressure (*p/p*_*o*_) was evaluated. Prior to the measurement, all samples were degassed.

Fourier transform infrared spectroscopy was used for the identification of characteristic functional groups, which are present on all prepared lignin-based spherical particles and pristine kraft lignin. The spectra were prepared with the use of Vertex 70 apparatus (Bruker Optics GmbH, Ettlingen, Germany) and the analysis was performed over the wavenumber range of 4,000 to 450 cm^−1^. Before analysis, 1 mg of dried sample and 250 mg of anhydrous potassium bromide were mixed and tableted in a still ring at a pressure of 10 MPa for 10 min. The spectrum of ionic liquid was performed with the use of attenuated total reflectance (ATR) spectroscopy. Single-reflection diamond ATR accessory (Platinum ATR, Bruker Optics GmbH) was used for conducting the analysis.

The elemental percentage content of carbon, hydrogen, nitrogen, and sulfur in all prepared samples and kraft lignin was evaluated with the use of a Vario EL Cube instrument (Elementar Analysensysteme GmbH, Langenselbold, Germany). The analysis is performed after high-temperature combustion in the oxygen atmosphere. Products pass through a catalyst in helium steam and then all obtained gasses are separated in an adsorption column and recorded with the use of a detector. 10 mg of every sample is weighed in tin foil packing, analysis is performed in triplicate, and the results are given to ±0.01%.

Electrophoretic mobility was also measured by using the Zetasizer Nano ZS instrument (Malvern Instruments Ltd., Malvern, United Kingdom), which was also equipped with an autotitrator (Malvern Instruments Ltd., Malvern, United Kingdom). During the measurement, the zeta potential is calculated and the electrokinetic curves can be determined for all prepared samples. Particle motion velocity is measured as a result of the application of variable electrical voltage. The laser light (λ = 633 nm) was dispersed by the moving particles and then it was recorded with the use of a detector. 10 mg of synthesized material was dispersed in 0.001 mol/L of sodium chloride electrolyte in an ultrasonic bath. The measurements were taken in a pH range from 2 to 10 and the pH of the suspension was adjusted with the use of 0.2 mol/L hydrochloric acid and 0.2 mol/L sodium hydroxide.

### 2.5 Antibacterial Activity

Antimicrobial activity tests were carried out using the standard shake flask method (Standard test method for determining the antimicrobial activity of antimicrobial agents under dynamic contact conditions). Gram-negative *Escherichia coli* (ATCC 10536) and gram-positive *Staphylococcus aureus* (ATCC 33592) were used in these studies. Frozen beads of examined species were thawed and subcultured onto nutrient broth (OXOID CM 0001) and incubated at 35 ± 2°C for 24 h. Cultures were centrifuged at 4,500 g for 10 min and cells were washed in deionized water. Cultures were resuspended in water and adjusted to 0.5 in the McFarland scale (108 cfu/ml) in McFarland Densitometer (Biosan). The materials (samples of lignin with 1-(propoxymethyl)-1H-imidazolium hydrogen sulfate in various weight ratios) were sterilized by autoclaving (121°C, 15 min). 0.1 g of analyzed sterile material were incubated with 100 ml of bacterial suspensions at 37°C and 230 rpm. The cell density of the suspensions before introducing material and after 0, 1, 4, and 24 h in contact with the material was determined using the pour plated method. Control samples were the bacterial suspensions in sterile water. These suspensions were decimally diluted in sterile physiological saline, plated on plate count agar, and incubated at 35 ± 2°C for 24–48 h to determine the number of surviving bacteria. The experiments were performed in duplicate.

## 3 Results and Discussion

### 3.1 Characteristics of Lignin-Based Spherical Particles (Lig-IL)

#### 3.1.1 Structural and Morphological Properties

**FIGURE 2:**
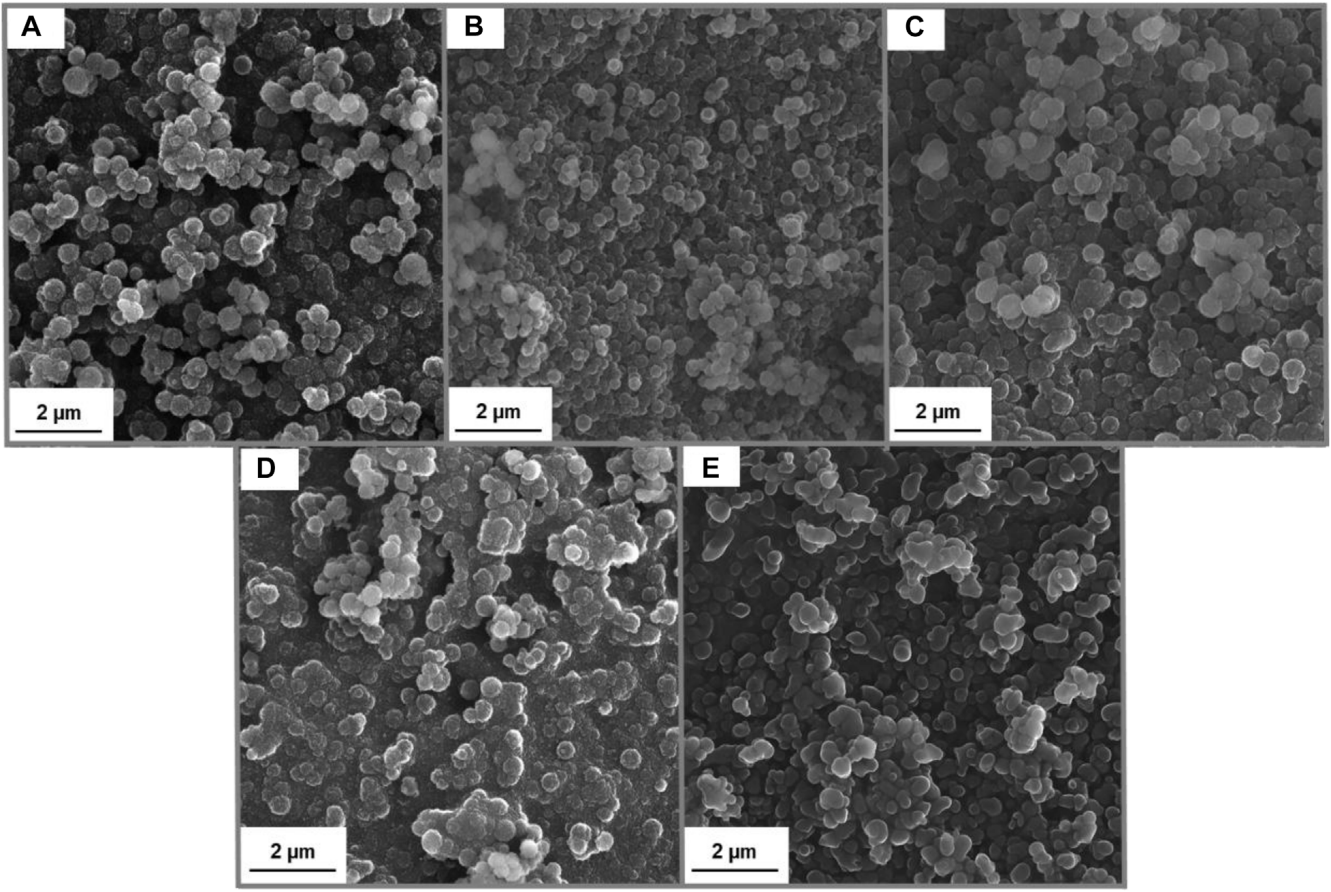
SEM images of Lig-IL (1:1) **(A)**, Lig-IL (4:3) **(B)**, Lig-IL (2:1) **(C)**, Lig-IL (4:1) **(D)**, and Lig-IL (10:1) **(E)**.

The main goal of this research was to prepare lignin-based spherical particles with the addition of hydrogen sulfate ionic liquid. Moreover, it was also tested, if the added amount of IL has a significant influence on the characteristic of obtained samples. Importantly, to evaluate the morphology and structure of prepared material SEM images and particle size distribution analyses were performed and the results were summarized in Figure 2; Table 1.

**TABLE 1:** Particle size distribution and polydispersity index for all prepared materials.

Sample	Particle Size distribution (nm)	Polydispersity index (PdI)
Lig-IL (1:1)	91–295	0.099
Lig-IL (4:3)	122–615	0.067
Lig-IL (2:1)	122–531	0.060
Lig-IL (4:1)	91–531	0.076
Lig-IL (10:1)	91–324	0.095
Kraft lignin	255-825; 3580-6,439	0.138

It can be observed, that the synthesis of lignin-based spherical particles was performed successfully and the materials with the required features were obtained. The amount of added ionic liquid may influence the formation of lignin-based particles. The samples prepared with a smaller amount of hydrogen sulfate ionic liquid show a higher ability to form aggregates and agglomerates and therefore the shape of particles is less firm especially for materials Lig-IL (4:1) (see Figure 2D) and Lig-IL (10:1) (see Figure 2E) than for samples prepared with a higher concentration of IL. Overall, it can be concluded, that ionic liquid interacted with kraft lignin and the process resulted in the preparation of small particles with well-developed microstructure and spherical shape.

All prepared particles were in sizes not exceeding 1 µm and according to the particle size distribution and polydispersity indices shown in Table 1, Lig-IL (1:1) exhibited the narrowest and the smallest particles in the range from 91 to 295 nm, which may also be confirmed with Figure 2A. Moreover, all products show low polydispersity indices below 0.100, which may indirectly confirm the correctness of the planned research. Particle size distribution analysis was also performed for untreated kraft lignin and it was observed, that the material exhibited the particle size distribution in two ranges from 255–825 nm to 3,580–6,439 nm and with a polydispersity index equal to 0.138. Comparing the results performed for all samples and kraft lignin, it may be observed that the use of ethyl alcohol allowed the partial dissolution (dispersion) of kraft lignin with some breakage of bonds, especially of the *β*- O -4 one. Therefore, the addition of hydrogen sulfate ionic liquid enabled the formation of homogenous spherical particles, which may form a stable suspension.

1-(propoxymethyl)-1H-imidazolium hydrogen sulfate was used as a surfactant and enabled the formation of lignin-based spherical particles, moreover, as has been mentioned before, the amount of added IL influences the shape of forming particles. Liu et al. (2019) dissolved different amounts of alkali lignin in a variety of imidazole ionic liquids and they observed, that with increased lignin concentration, the obtained spherical particles exhibited a larger diameter, than the particles prepared with lower biopolymer concentration. Cao et al. (2021) prepared spherical particles with the use of the spray drying method. They controlled the shape and size of particles by adjusting the concentration of potassium hydroxide (KOH). With the increased addition of KOH, the change in density, surface tension, and viscosity was observed, therefore the particles exhibited different structures, from dense to hollow. Similar results were also presented by Kitamoto et al. (2022), who used sodium hydroxide in various ratios and it was also observed that the morphology of spherical particles changed from dense to hollow.

#### 3.1.2 Structural and Morphological Properties

Prepared lignin-based spherical particles underwent porous structure analysis. The BET surface area (*A*_*BET*_), mean size (*S*_*p*_) as well as the total volume of pores (*V*_*p*_) were evaluated. The data are shown in Table 2 and it can be concluded that the BET surface area values, the pore size, and volume were influenced by the modification of lignin. It has been evaluated, that kraft lignin exhibits low values of porous structure parameters including a low surface area value of 1 m^2^/g, pore sizes around 12 nm, and a total volume of pores of 0.011 cm^3^/g (Szalaty et al., 2018; Fila et al., 2022).

**TABLE 2:** Porous structure parameters of all prepared samples.

Sample	Structural properties		
	*A*_*BET*_ (m^2^/g)	*Vp* (cm^3^/g)	*Sp* (nm)
Lig-IL (1:1)	15	0.006	2.06
Lig-IL (4:3)	14	0.006	2.24
Lig-IL (2:1)	17	0.008	2.13
Lig-IL (4:1)	14	0.005	2.06
Lig-IL (10:1)	12	0.003	2.05
Kraft lignin	1	0.011	12.02

After modification of lignin with the use of hydrogen sulfate ionic liquid to prepare spherical particles, the increase of surface area from 1 m^2^/g to 12–17 m^2^/g was detected. Moreover, the mean size of pores decreased from 12.02 nm to ca. 2 nm for all prepared samples. There are some changes in terms of the mean sizes of pores. The largest ones were observed for Lig-IL (4:3) and Lig-IL (2:1) with the values of 2.24 and 2.13 nm, respectively. Moreover, the value of the total volume of pores is also the highest for these two samples. The lowest values of mean size and total volume of pores were observed for Lig-IL (10:1), which contained the smallest amount of ionic liquid, compared to other samples. The best result of surface area was observed for Lig-IL (2:1) sample at around 17 m^2^/g. It may be related to the best ratio of lignin and ionic liquid, which were used for the preparation of lignin-based spherical particles. It has been shown, that lignin-based spherical particles can be prepared with the use of hydrogen sulfate ionic liquid and moreover, the change in morphology of lignin material enabled the change in the porous structure of all prepared materials. Similar results were obtained by Klapiszewski et al. (2017) They used hydrogen sulfate ionic liquids for dissolution and modification of kraft lignin. After the procedure, obtained material exhibited a surface area of around 14–18 m^2^/g, which may indicate, that the addition of hydrogen sulfate ionic liquid improves the values of surface area, which may be useful for potential application routes. Chen et al. (2022) prepared aminated lignin particles and they observed that during the modification process the lignin surface area was enlarged almost ten times compared to the pristine biopolymer. They also presented that the total pore volume of the sample also increased ten times. Lotfy and Basta (2022) synthesized lignin nanoparticles and it was also highlighted, that the prepared material showed the surface area values increased ten times compared to pristine kraft lignin.

#### 3.1.3 Chemical Structure Characteristics

**FIGURE 3:**
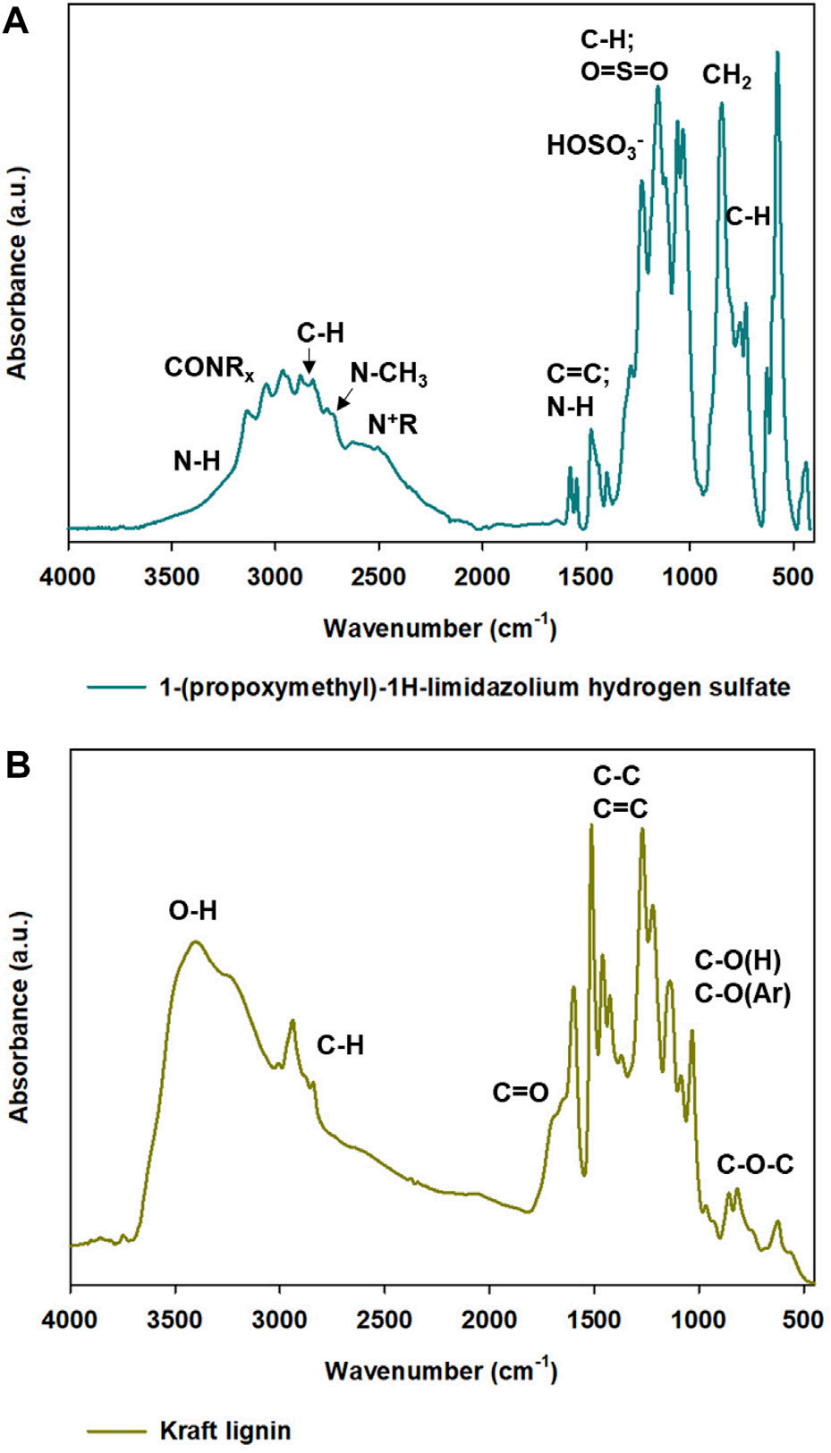
ATR-FTIR spectra of precursors used for synthesis of lignin-based spherical particles: 1-(propoxymethyl)-1H-imidazolium hydrogen sulfate **(A)** and kraft lignin **(B)**.

**FIGURE 4:**
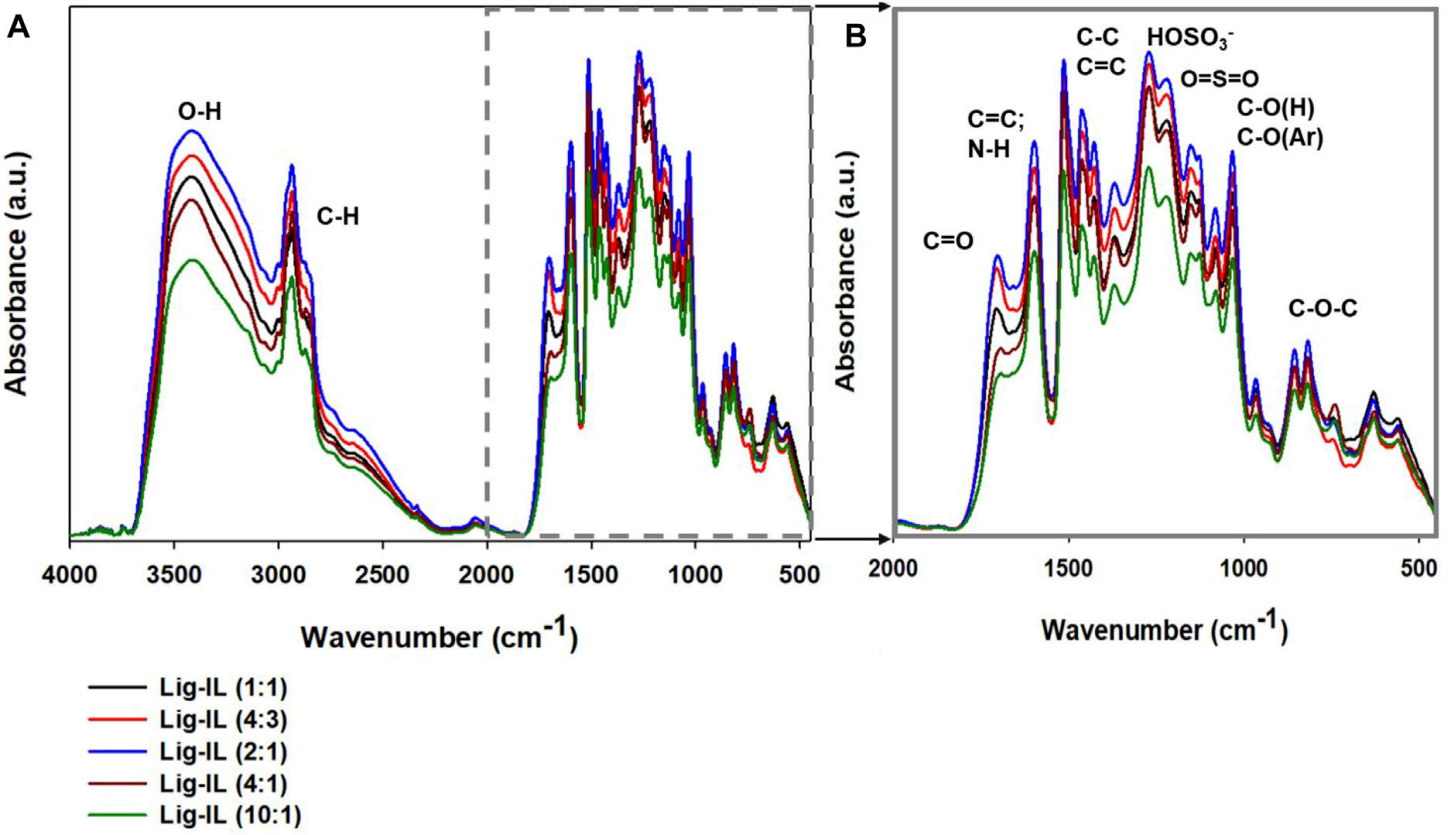
FTIR spectra of all prepared materials **(A, B)**, shown in two wavenumber ranges.

Fourier transform infrared and attenuated total reflectance spectra were performed to determine the characteristic bands in kraft lignin, hydrogen sulfate ionic liquid, and synthesized samples. Throughout the research, different ratios of lignin and IL were used for the formation of particles. The purpose of ionic liquid was to act as a soft-template and in specific conditions form lignin-based spherical particles. FTIR spectra of all prepared materials were performed to evaluate the correctness of the proposed method and to examine, which functional groups could be detected on the surface of the obtained materials. The FTIR spectra of kraft lignin and ATR spectra of hydrogen sulfate ionic liquid are presented in Figure 3 and FTIR spectra for all prepared samples are presented in Figure 4.

In the ATR spectrum of 1-(propoxymethyl)-1H-imidazolium hydrogen sulfate the functional groups of the used ionic liquid are visible. There are the characteristic bands, which are related to cation including the peak occurring at wavenumber 3300 cm^−1^ corresponds to the stretching vibrations of the -NH group (Abdelhamid et al., 2020). Moreover, there is a wide band that is responsible for stretching vibrations of -CH from 2,970 cm ^−1^ to 2,900 cm^−1^ and of N-CH_3_ at the maximum of wavenumber at 2800 cm^−1^. There is also a presence of characteristic functional groups which are related to the anion, including HOSO_3_^−^ (1250 cm^−1^) and O=S=O (1164 cm^−1^) (Gupta et al., 2017; Nurdin et al., 2021).

FTIR spectra of all prepared products (shown in Figure 4A) reflect the structure of pristine kraft lignin. In all lignin-based materials, there are characteristic bands with are connected to pristine kraft lignin and also the used hydrogen sulfate ionic liquid. The wide band observed from 3,600 cm^−1^ to 3100 cm^−1^ corresponds to the stretching vibrations of -OH groups. Importantly, in comparison to the spectrum of kraft lignin, all materials prepared with IL exhibit a slightly different shape of this broad peak. The shape of the hydroxyl peak in kraft lignin spectra indicates that there are a lot of alcoholic and carboxylic hydroxyl groups, while in all prepared samples there are more phenolic hydroxyl functional groups. The switch of hydroxyl groups may be associated with partial oxidation to carbonyl groups. Moreover, a sharp peak occurring at 3,000 cm^−1^ corresponds to the stretching vibrations of -CH groups and may also confirm partial depolymerization of kraft lignin. The addition of hydrogen sulfate ionic liquid enabled several changes in lignin-based spherical particles compared to pristine biopolymer and there are some visible differences between particular spectra. Important changes may be observed at the wavenumber with a maximum at 1,720 cm^−1^, namely a band from the carbonyl group was registered. Naturally, kraft lignin exhibits a weak carbonyl group, which is mostly covered by the signals from the aromatic skeleton, therefore there are small amounts of carbonyl functional groups on pristine kraft lignin. The addition of ionic liquid and its interaction with kraft lignin intensified the peak from carbonyl groups significantly. The carboxylation of kraft lignin is very beneficial, because, as was presented in another publication, the increasement of carbonyl functional groups enables novel application such as electrochemistry (Lam et al., 2017; Klapiszewski et al., 2018b; Yang et al., 2021). It was observed, that the addition of lignin and hydrogen sulfate ionic liquid in a ratio of 2:1 results in the sharpest and intensive peak from the carbonyl group. On the other hand, lignin and the ionic liquid ratio of 10:1 show low efficiency, and the carbonyl group was registered with the lowest intensity of the carbonyl band. It can be concluded, that addition of a specific amount of ionic liquid may have an influence on the presence of a variety of functional groups with different intensities. It may also be confirmed that the best ratio of lignin to ionic liquid is 2:1 according to FTIR spectra. It may be linked to partial depolymerization and oxidation of kraft lignin with the use of ionic liquid during the formation of lignin-based spherical particles and the ratio of 2:1 may act the most effectively. There is also a weak signal from the -N + R group at a wavenumber of 2600 cm^−1^ and quite strong ones corresponding to the HOSO_3_^−^ group and O=S=O one at wavenumbers of 1220 cm^−1^ and 1145 cm^−1^, respectively. The described signals are related to the presence of hydrogen sulfate ionic liquid in the presence of lignin-based spherical particles, which indirectly confirms the incorporation of IL in the biopolymer structure.

As has been mentioned before, the different ratios of lignin and hydrogen sulfate ionic liquid were used for the preparation of lignin-based spherical particles. According to FTIR spectra analysis, it was observed that the prepared materials exhibit functional groups, that are characteristic of both precursors. There are some differences in intensity depending on the used ratio of lignin and ionic liquid and it may be connected to the interaction of ionic liquid and biopolymer, especially the ability of kraft lignin for partial depolymerization and oxidation.

#### 3.1.4 Elemental Analysis

The elemental content of carbon, hydrogen, sulfur, and nitrogen in lignin-based spherical particles, as well as in kraft lignin is presented in Table 3. Kraft lignin contains carbon, hydrogen, oxygen, as well as other elements such as nitrogen or sulfur. Some of the elements may be present in the lignin sample regarding its origin, growth, and minerals. Sulfur content may also vary through the preparation method (Ahmad et al., 2020; Yan et al., 2020). It was observed, that there is a similar level of analyzed elements in every obtained sample. There is no visible correlation between the contents of elements in prepared lignin-based spherical particles and the amount of ionic liquid, which was used for the synthesis of products. In comparison to kraft lignin, there was a percentage increase in every element. It may be concluded, that ionic liquid was chemically combined with lignin and the preparation of spherical particles was performed successfully. In every synthesized sample, the nitrogen and increased value of sulfur were detected and therefore the presence of cation and anion of ionic liquid is confirmed in the structure of spherical particles. Moreover, the increased carbon and hydrogen content showed that the ionic liquid was combined with kraft lignin effectively. As has been mentioned before, the amount of added lignin has no significant impact on the elemental content of prepared materials. It may be connected to the fact, that only a limited amount of kraft lignin can be chemically bounded with ionic liquid, but on the other hand, the ionic liquid content influences the preparation of spheres, as was shown in 2.1.1. Structural and Morphological Properties paragraph.

**TABLE 3:** Elemental analysis of all prepared materials.

Sample	Elemental content (%)			
	N	C	H	S
Lig-IL (1:1)	0.8	48.5	8.9	3.5
Lig-IL (4:3)	0.7	47.5	9.2	3.7
Lig-IL (2:1)	0.8	49.5	9.2	3.4
Lig-IL (4:1)	1.0	48.3	8.9	3.6
Lig-IL (10:1)	0.9	49.0	8.4	3.4
Kraft lignin	-	36.1	5.7	2.7

#### 3.1.5 Zeta Potential

**FIGURE 5:**
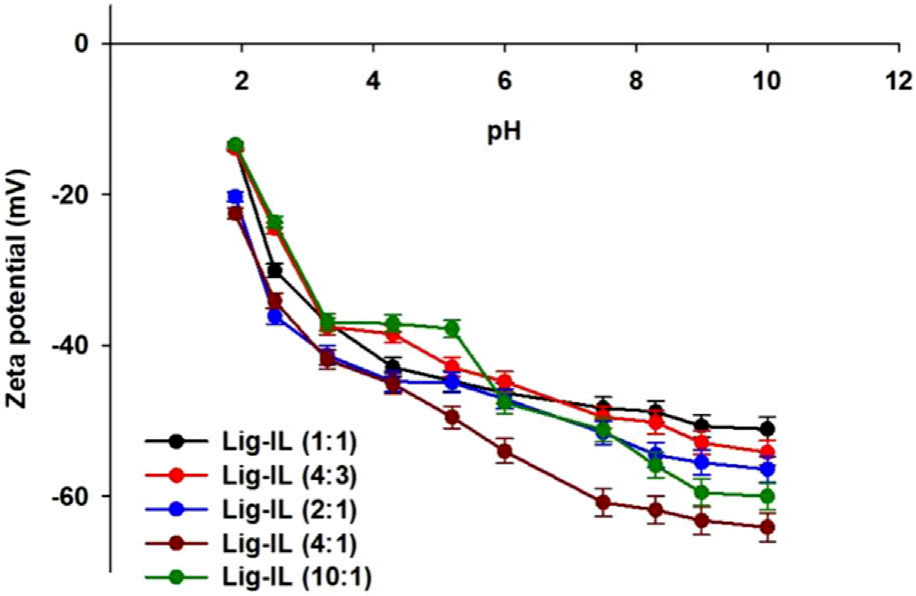
Zeta potential *vs*. pH for all prepared samples.

The electrokinetic evaluation of obtained lignin-based spherical particles was performed during the physicochemical evaluation and it was presented in Figure 5 and also the results were summarized in Table 4. The analysis enables the introduction of the correlation between zeta potential and pH and therefore the evaluation of electrokinetic stability of prepared spherical particles and the determination of the composition of functional groups present on the surface of the material can be performed. Electrokinetic stability is linked to the ionization of specific functional groups, mostly hydroxyl and carbonyl ones. The material is considered an electrokinetic stable, when zeta potential values are above 30 mV or under –30 mV.

**TABLE 4:** Zeta potential values for all materials in different pH values.

Sample	pH									
	1.9	2.5	3.3	4.3	5.2	6.0	7.5	8.3	9.0	10.0
	Zeta potential value (mV)									
Lig-IL (1:1)	−13.7 (±0.4)	−30.1 (±0.9)	−36.9 (±1.1)	−42.9 (±1.3)	−44.7 (±1.3)	−46.3 (±1.4)	−48.3 (±1.4)	−48.8 (±1.5)	−50.7 (±1.5)	−51.7 (±1.5)
Lig-IL (4:3)	−13.9 (±0.4)	−24.5 (±0.7)	−37.5 (±1.1)	−38.5 (±1.2)	−42.9 (±1.3)	−44.8 (±1.3)	−49.5 (±1.4)	−50.2 (±1.4)	−52.9 (±1.5)	−54.2 (±1.5)
Lig-IL (2:1)	−20.3 (±0.6)	−36.1 (±1.1)	−41.3 (±1.2)	−44.8 (±1.3)	−44.9 (±1.3)	−47.1 (±1.4)	−51.6 (±1.5)	−54.5 (±1.6)	−55.5 (±1.7)	−56.4 (±1.7)
Lig-IL (4:1)	−22.5 (±0.7)	−34.1 (±1.0)	−41.9 (±1.3)	−45.1 (±1.4)	−49.5 (±1.5)	−54.0 (±1.6)	−60.8 (±1.8)	−61.8 (±1.9)	−63.2 (±1.9)	−64.1 (±1.9)
Lig-IL (10:1)	−13.4 (±0.4)	−23.7 (±0.7)	−36.9 (±1.1)	−37.1 (±1.1)	−37.8 (±1.1)	−47.6 (±1.4)	−51.2 (±1.5)	−55.9 (±1.7)	−59.5 (±1.8)	−60.0 (±1.8)

Kraft lignin was used for the preparation of biopolymer-based spherical particles and as it was confirmed in several different research, the material exhibit a negative charge in the whole studied pH range, therefore it shows no isoelectric point. The material exhibits good electrokinetic stability at pH above 6 and the lowest zeta potential value was recorded at around –35 mV at pH 10. Similar results were presented by Chen *et al.* They showed that kraft lignin exhibited negative zeta potential value in pH range from 3 to 10. Moreover, the material was colloidally stable around pH 5 (Chen et al., 2022). Zeta potential was determined in a pH range from 2 to 10 and it was concluded, that prepared lignin-based spherical particles exhibit several different functional groups on the surface of the material. Lignin-based spheres are electrokinetically stable over almost the entire pH and the negative values of zeta potential show that the surface charge of synthesized particles is also negative. There are no significant differences between the obtained curves for all materials. Most of the samples exhibited electrokinetic stability around pH 3, but samples Lig-IL (2:1) and Lig-IL (4:1) were electrokinetically stable at pH 2. The added amount of ionic liquid has no significant impact on the zeta potential values of every pre-pared sample. There are several small differences between prepared samples. Lig-IL (4:1) showed the lowest valuesthe of zeta potential parameter therefore it may be concluded that it has the highest content of functional groups which are suitable for ionization. The analysis of the lowest values of zeta potential of Lig-IL (4:1) material may lead to the assumption that the negatively charged material will attract positively charged particles. Lig-IL (4:1) may also show the highest colloidal stability in a variety of reaction conditions. Moreover, all remaining samples showed zeta potential values from –13.4 mV to –60.0 mV indicating great electrochemical stability. Similar results were presented by Liu *et al.* They synthesized lignin-based nanospheres, which were also electrokinetically stable in pH range from 4 to 12, therefore its general great colloidal stability enables the possibility of industrial use of prepared material (Liu et al., 2019). Yan *et al.* prepared kraft lignin nanospheres with excellent stability. Particles exhibited high negative zeta potential (around –50 mV at pH 7, depending on the preparation method). The negative values may prevent aggregation through dual electric repulsion (Yan et al., 2020). An *et al.* modified lignin with the use of 3-dimethylamino-1-propyl chloride hydrochloride and 1,3-propanesultone. They also observed negative values of zeta potential (below –30 mV) for most samples (An et al., 2021). Moreover, negative surface charges were also observed for materials prepared by Cao *et al.* They presented that higher addition of KOH changes the zeta potential values from –52.8 mV to –72.7 mV) to retain the spherical shape through electrostatic repulsion (Cao et al., 2021).

It can be observed, that all prepared lignin-based spherical particles show the ability to dissociate because of the presence of many functional groups. The addition of ionic liquid might increase the quantity of protonable groups including carboxylic, sulfonic, hydroxyl, and carbonyl groups. These functional groups can form anionic forms and therefore the overall system is more stable.

As has been mentioned and proved before, prepared particles exhibit a variety of functional groups. It has been shown, that phenolic and aliphatic hydroxyl, carboxyl, and methoxy groups are biologically active and therefore the material may show antioxidant properties (Lupoi et al., 2015; Kaur et al., 2017; Lobo et al., 2021). Moreover, the antibacterial activity is related to the origin of a sample and the presence of different functional groups, especially ones, that contain oxygen in its structure (Alzagameem et al., 2019). Prepared samples also contain sulfur in their structure which may also enhance the antibacterial and antimicrobial properties of materials (Tao et al., 2021). The prepared particles exhibited the presence of functional groups; therefore, they were further tested for their antibacterial properties towards specific bacteria.

### 3.2 Antibacterial Activity

**FIGURE 6:**
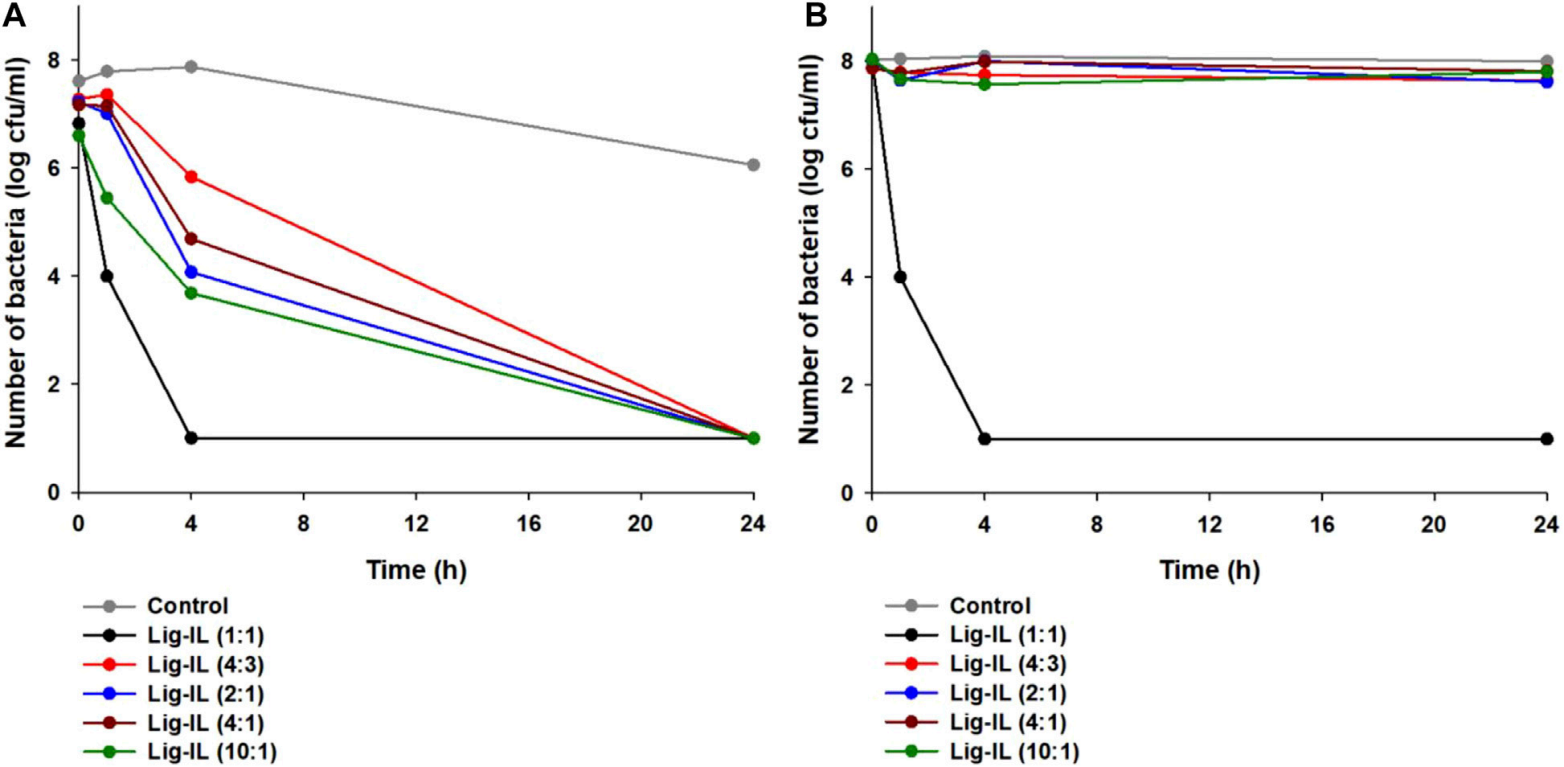
Antibacterial activity of examined materials against *Staphylococcus aureus* **(A)** and *Escherichia coli* **(B)**.

The antimicrobial activity of tested materials was examined against two representative strains of bacteria, gram-negative *Escherichia coli* and gram-positive *Staphylococcus aureus*, using the shake flask method, and the results are presented in Figure 6. Better results from antibacterial activity testing were obtained in the case of gram-positive *Staphylococcus aureus* (see Figure 6A).

After 1 h of contact of bacteria with lignin-based spherical particles with the addition of hydrogen sulfate ionic liquid in proportion 1:1, a 50% reduction in the number of microorganisms was observed. After the next 4 h, the number of bacteria decreased from the initial 8 log cycles to 1 log and remained at this level until the end of the experiment. Fabricated lignin-based spherical particles with 1-(propoxymethyl)-1H-imidazolium hydrogen sulfate in other proportion indicated weaker antimicrobial activity, but after 24 h of contact the tested materials with *Staphylococcus aureus* cells the number of microorganisms has lowered to the level of 1 log cycle. The investigations of antibacterial activity of tested materials against gram-negative *Escherichia coli* showed that only one was effective–lignin-based spherical particles with the addition of hydrogen sulfate ionic liquid in proportion 1:1 (see Figure 6B). Interestingly, its effectiveness was almost identical to that against gram-positive *Staphylococcus aureus*. The differences in the antibacterial activity against both strains may be related to the structure of the cell walls of bacteria. *S. aureus* shows no external lipid membrane with a peptigoglycan cell wall. On the other hand, *E. coli* bacteria exhibit lipopolysaccharide membrane which protects the external wall of a strain (Huang et al., 2022). It has been proven, that the antibacterial activity is related to the stability and the hydrophilic or hydrophobic nature of examined material as well as the structure of the material (Saif et al., 2021; Zafar et al., 2021; Wu et al., 2022). It was noted, while performing zeta potential analysis, that all prepared materials show excellent suspension stability in aqueous solutions in a variety of pH ranges, therefore prepared materials may be suitable for application as antimicrobial agents, especially for gram-positive bacteria. No antimicrobial activity of kraft lignin was observed (data not shown). Interestingly, the mechanism of antibacterial activity of prepared samples is not fully known yet, however; it has been reported that there are reactive oxygen species on the surface of the lignin sphere, therefore the oxidative stress may be induced, and the cell membrane of bacteria can be damaged (Yun et al., 2021).

Lignin- or cellulose-based micro- and nanomaterials capable of encapsulating active substances have been of great interest to scientists in recent times. These nanocarriers may be loaded by medicines (e.g., antibiotics), natural bioactive products (e.g., extracts from medicinal plants), porphyrins, silver ions or metal oxides (Bazant et al., 2014; Klapiszewski et al., 2015; Li et al., 2020; Maldonado-Carmona et al., 2020; Paudel et al., 2021; Sánchez-Hernández et al., 2022). This allows to obtain antimicrobial activity of such materials. In our investigations lignin-based spherical particles with the use of ionic liquid - of 1-(propoxymethyl)-1H-imidazolium hydrogen sulfate in various weight ratios was used. Very good efficiency against *Staphylococcus aureus* and satisfaction against *Escherichia coli* was achieved. Differences in the action of tested materials on gram-positive and gram-negative bacteria are usually due to the structure of membranes and the cell wall. gram-negative *Escherichia coli* has an outermost lipopolysaccharide layer, thin peptidoglycan layer, and phospholipid bilayer (monolayer in gram-positive *Staphylococcus aureus*) (Kuhn et al., 2015). The mechanism of antibacterial action of tested materials could be explained by the adsorption of bacteria onto lignin-based spherical particles’ surfaces and the destruction of the outer membrane and cell wall of bacteria by the ionic liquid, which is crucial for bacterial cell death. These antimicrobial properties of obtained lignin-based spherical particles with the use of ionic liquid—1-(propoxymethyl)-1H-imidazolium hydrogen sulfate give the possibility of their use in the industries requiring antibacterial action of the material, for example sanitary, hygienic or biomedical applications.

## 4 Conclusion

In the present study, kraft lignin was combined with hydrogen sulfate ionic liquid with the use of a self-templating method. For the preparation of spheres, different weight ratios of kraft lignin and IL were used. Obtained materials were tested morphologically and physiochemically. Analysis of SEM images and particle size distribution showed that the addition of ionic liquid enabled the formation of spherical particles with good homogeneity and narrow sizes. The porous structure of presented particles was also evaluated, and it was concluded that the preparation of novel material increased the value of surface area (from 1 m^2^/g to 14–17 m^2^/g) with decreased sizes of pores in their structure. With the ATR-FTIR analysis, it was evaluated that obtained particles inherited most of the functional groups from kraft lignin, but the specific bonds from IL were visible; therefore, it can be concluded that ionic liquid was successfully incorporated into the lignin structure. The presence of reactive functional groups including hydroxyl, carboxylic, and carbonyl was detected. The presence of carbon, sulfur, nitrogen, and hydrogen was confirmed with the use of the elemental analysis and the increase of all elements was presented, which also may be related to the addition of ionic liquid into the system. All particles were stable in acidic conditions (above pH 3) and exhibited negative zeta potential values in the analyzed pH range.

The prepared characteristic of lignin-based spherical particles encouraged testing of the antibacterial properties of produced samples. It was proven, that all materials exhibited the antibacterial activity against gram-positive *Staphylococcus aureus*. The best results were observed for the Lig-IL (1:1) sample, which contained the highest amount of ionic liquid during its preparation. Particles were also tested for antibacterial activity against gram-negative *Escherichia coli*. Interestingly, only the Lig-IL (1:1) material exhibited almost identical activity against this bacterium as for gram-positive one. It may be concluded, that the highest concentration of ionic liquid enhanced the antibacterial activity of prepared materials.

## Data Availability

The original contributions presented in the study are included in the article/supplementary material, further inquiries can be directed to the corresponding author.
